# Composition of the Spruce Budworm (*Choristoneura fumiferana*) Midgut Microbiota as Affected by Rearing Conditions

**DOI:** 10.1371/journal.pone.0144077

**Published:** 2015-12-04

**Authors:** Mathieu Landry, André M. Comeau, Nicolas Derome, Michel Cusson, Roger C. Levesque

**Affiliations:** 1 Institut de biologie intégrative et des systèmes (IBIS) et Faculté de médecine, Université Laval, Québec, Canada; 2 CGEB-Integrated Microbiome Resource (CGEB-IMR), Department of Pharmacology, Dalhousie University, Halifax, Nova Scotia, Canada; 3 Institut de biologie intégrative et des systèmes (IBIS) et Faculté des sciences et de génie, Université Laval, Québec, Canada; 4 Natural Resources Canada, Laurentian Forest Centre, Québec, Canada; Ghent University, BELGIUM

## Abstract

The eastern spruce budworm (*Choristoneura fumiferana*) is one of the most destructive forest insect pests in Canada. Little is known about its intestinal microbiota, which could play a role in digestion, immune protection, communication and/or development. The present study was designed to provide a first characterization of the effects of rearing conditions on the taxonomic diversity and structure of the *C*. *fumiferana* midgut microbiota, using a culture-independent approach. Three diets and insect sources were examined: larvae from a laboratory colony reared on a synthetic diet and field-collected larvae reared on balsam fir or black spruce foliage. Bacterial DNA from the larval midguts was extracted to amplify and sequence the V6-V8 region of the 16S rRNA gene, using the Roche 454 GS-FLX technology. Our results showed a dominance of Proteobacteria, mainly *Pseudomonas* spp., in the spruce budworm midgut, irrespective of treatment group. Taxonomic diversity of the midgut microbiota was greater for larvae reared on synthetic diet than for those collected and reared on host plants, a difference that is likely accounted for by several factors. A greater proportion of bacteria from the phylum Bacteroidetes in insects fed artificial diet constituted the main difference between this group and those reared on foliage; within the phylum Proteobacteria, the presence of the genus *Bradyrhizobium* was also unique to insects reared on artificial diet. Strikingly, a Bray-Curtis analysis showed important differences in microbial diversity among the treatment groups, pointing to the importance of diet and environment in defining the spruce budworm midgut microbiota.

## Introduction

Insects can derive diverse benefits from their intestinal microbiota. The best example is the termite, whose digestive track is specifically adapted for colonization by various microbes essential to the digestion of wood biomass [1]. The microbial community of the termite intestinal microbiota consists largely of Proteobacteria, Spirochetes, Bacteroidetes and Gram-positive bacteria with a low G+C content [2]. In contrast, bacterial symbionts are apparently not essential for digestion of the plant cell wall in many other insect species that secrete digestive enzymes encoded in their own genomes [3]. However, there are other benefits associated with midgut bacteria, including enhanced resistance to pathogens [4], promotion of intestinal epithelium development [5], neutralization of toxins [6] and production of chemical communication molecules [7]. Lepidopteran insects feed mostly on plants and possess a very simple digestive system devoid of any known adaptation for the maintenance of microbes. The majority of bacteria composing their microbiota are acquired from their food plants [8], making diet an important factor in microbiota composition. The microbiota has no defined role in the Lepidoptera, but it is often dominated by Enterobacteriaceae, which could play a role in carbohydrate metabolism [9].

Each year in Canada, defoliating insects are responsible for important timber volume losses for the forest industry. Controlling these pests is a significant challenge and scientists are constantly looking for novel strategies that are more cost-efficient and/or eco-friendly. As chemical pesticides are no longer looked upon favorably for use in forest pest management, molecular and biological targets that can be perturbed in a pest-specific manner have become the focus of many current research efforts aimed at developing alternative pest-control tools. The intestinal flora of these insects could be one such target, particularly where it plays a significant role in host health.

It is in this context that we undertook the present work on the midgut microbiota of the spruce budworm, *Choristoneura fumiferana*, a major pest of spruce and fir in Canada. Our primary objective was to conduct a survey of this insect’s gut microflora using a next-generation sequencing (NGS) approach. A pioneering study that examined the intestinal microbiota of *C*. *fumiferana* indicated a dominance of *Enterococcus* and *Staphylococcus* bacteria among microorganisms that could be cultured from its midgut, and showed that antibiotics present in a synthetic diet [10] were not potent enough to eliminate gut bacteria at the concentrations used [11]. Another study also showed that intestinal juices from larvae fed balsam fir foliage could inhibit bacterial growth [12], suggesting that the host plant could have a negative impact on the budworm midgut microflora. For this reason, the second objective of our study was to conduct a preliminary assessment of the effect of rearing conditions, including diet (synthetic, black spruce, balsam fir) and insect sources (laboratory colony *versus* field-collected) on budworm midgut microbiota composition. Our results point to a significant effect of food and insect source on the taxonomic diversity and structure of the budworm midgut microbiota.

## Materials and Methods

Insects were collected by government scientists in permanent research plots located on crown land; no permission was therefore required. The sampling was considered non-destructive and did not involve protected or endangered species.

### Experimental insects

For this study we used 36 *C*. *fumiferana* larvae, distributed evenly among three experimental groups (food sources). For the first group, larvae were obtained from Insect Production Services (Natural Resources Canada, Sault Ste. Marie, Canada) as post-diapause second instars and were reared at room temperature on a synthetic wheat germ diet containing Aureomycin at a level of 0.56% [10]. The other two experimental groups were made up of larvae collected in the field near Baie-Comeau, Quebec, as third and fourth instars feeding on balsam fir (*Abies balsamea*) or black spruce (*Picea mariana*). These larvae were held in plastic containers and reared on young shoots from the same trees they were collected on until they reached the sixth instar. All larvae were sacrificed as mid-sixth instars for midgut dissection.

### Midgut dissections and DNA extraction

To remove surface microbes, larvae were briefly dipped in 70% ethanol prior to midgut dissection. Tissue was excised using ethanol-sterilized fine forceps and opened up longitudinally to gently remove coarse, undigested material remaining at the time of dissection. The midguts were homogenized individually in 1.5 mL microfuge tubes containing 200 μL of sterile phosphate-buffered saline (PBS) using sterile plastic pestles. Genomic DNA was extracted from the midguts following the Gram-positive protocol of the DNeasy Blood and Tissue Kit (Qiagen).

### Choice of primers for Roche 454 pyrosequencing

We used primers designed by Comeau et al. (2011) that target the V6-V8 region of bacterial 16S rRNA. In preliminary tests using spruce budworm midgut DNA, amplicons from this region provided the best taxonomic resolution. Forward (F) primers included Roche’s A adaptor and MIDs (‘‘multiplex identifiers”) in the following arrangement: 5’-[A-adaptor]+[MID1 to 10]+[specific F primer]-3’. Reverse (R) primers included Roche’s B adaptor in the following arrangement: 5’-[B-adaptor]+[specific R primer]-3’. Full primer sequences are listed in S1 Table.

### Pyrosequencing of the 16S rRNA gene

Amplification of the 16s rRNA gene, equimolar pooling and sequencing was performed at the Plateforme d’analyses génomiques (IBIS, Université Laval, Quebec City, Canada) following the procedure described in Comeau et al. [13]. Briefly, the extracted DNA was amplified by PCR using the aforementioned fusion primers, followed by amplicon purification. Then, for each of the three treatments, equimolar amounts of the 12 replicate amplicons were pooled and loaded onto 1/8th of a sequencing plate and sequenced on a Roche 454 GS-FLX + platform. The sequences were deposited in SRA under accession number SRP065845—PRJNA300845.

### Sequence pre-processing

We used mothur v.1.30.2 [14] to curate the dataset so as to retain only reads with a length between 350 and 450 bps, without Ns and with 100% correct F primer sequences. Sequences were aligned using mothur and chimeras were removed by alignment of a query sequence with two parent sequences using UCHIME [15]. Sequences classified as originating from chloroplasts were also removed using mothur. Finally, samples (individual larvae) were normalized by random down-sampling to 2460 reads each (the smallest number of final, quality-controlled reads among the samples).

### Data analysis

Mothur was used for all the subsequent analyses. The curated reads were first clustered at a 97% identity cutoff in order to generate operational taxonomic units (OTUs) that roughly correspond to individual bacterial species [14]. Rarefaction curves (α-diversity) for each experimental group were generated and β-diversity analyses (among samples and treatments) were done by calculating Bray-Curtis distances and Sørensen similarity values (abundance-based version ${\hat{L}}_{abd}$ suggested by Chao et al. [2005]). OTUs were classified using the GreenGenes97 reference database as modified by Comeau et al. (2011), and selected sequences were manually compared to the NCBI nr database using the BLASTn algorithm [16].

### Statistical analysis

The non-parametric Mann-Whitney and Kruskal-Wallis tests were used to assess the statistical significance of differences among treatments when the data did not fulfill the conditions of normality. In all other cases, ANOVA, F- and t-tests (equal or unequal variance, as the case may be) were used. All statistical analyses were carried out using the software PAST (folk.uio.no/ohammer/past/).

## Results

### Variation in richness among microbiotas (α-diversity)

**Fig 1:**
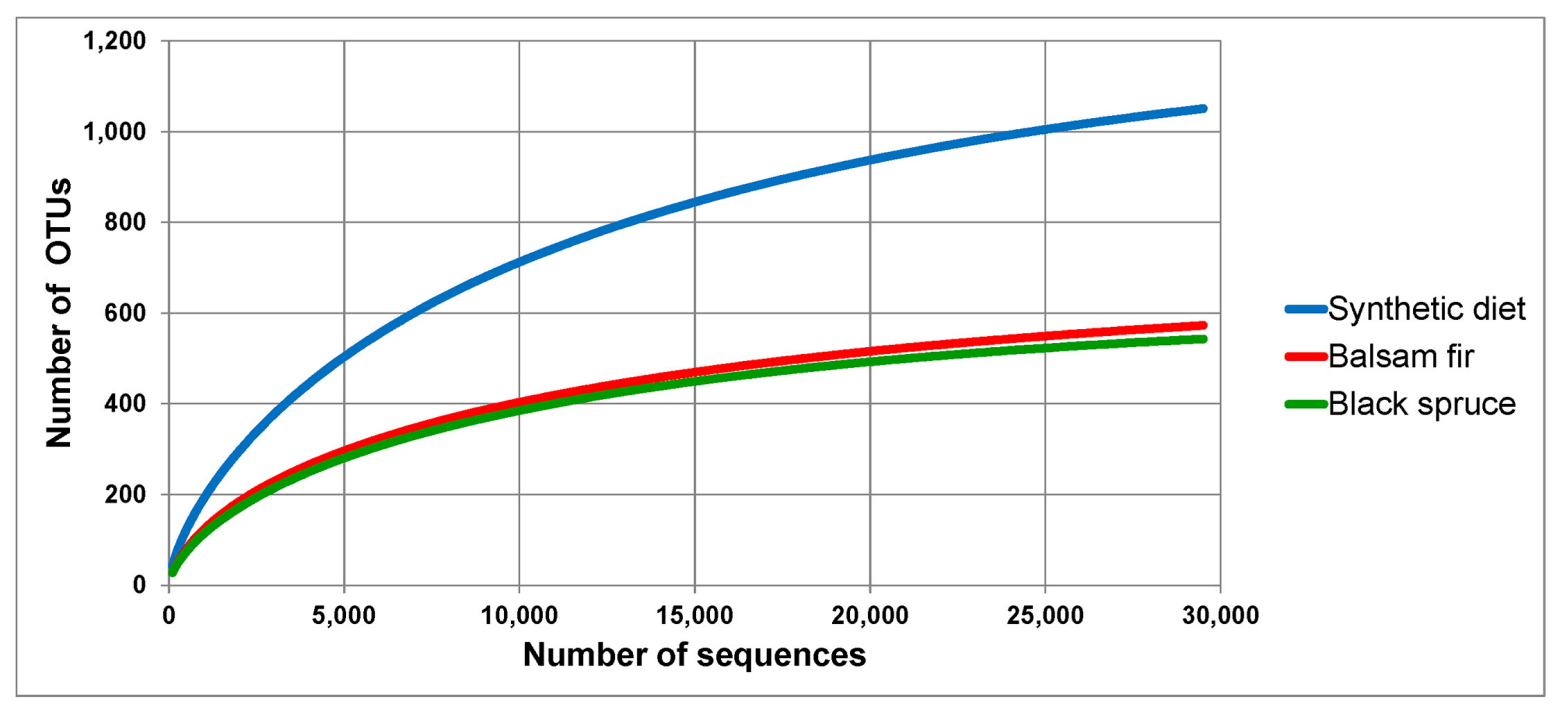
OTU rarefaction curves for each experimental group. Each group represents the aggregate of 12 individuals, at 2,460 reads each (total 29,520 reads per group). Note that the balsam fir and black spruce curves have overlapping 95% confidence intervals (i.e., not significantly different).

For each of the three treatment groups, the OTU rarefaction curves began to plateau by the time all reads had been analyzed, indicating that the sequencing depth was sufficient to capture most of the biodiversity found in the larval midguts (Fig 1). Interestingly, far more OTUs were detected in larvae reared on a synthetic diet containing Aureomycin (>1000) than in larvae collected in the field on either balsam fir or black spruce (<600).

### Overall composition of spruce budworm midgut microbiota

**Fig 2:**
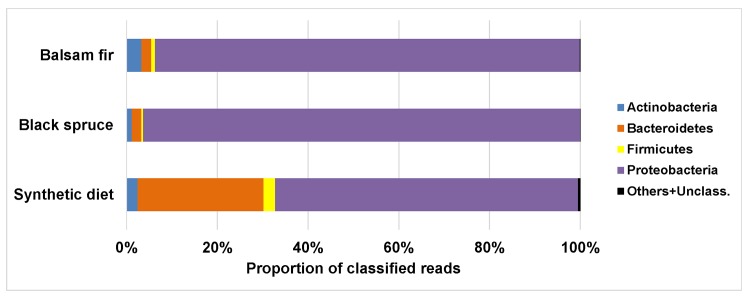
Taxonomic distribution of reads at the phylum level for each experimental group.

**Fig 3:**
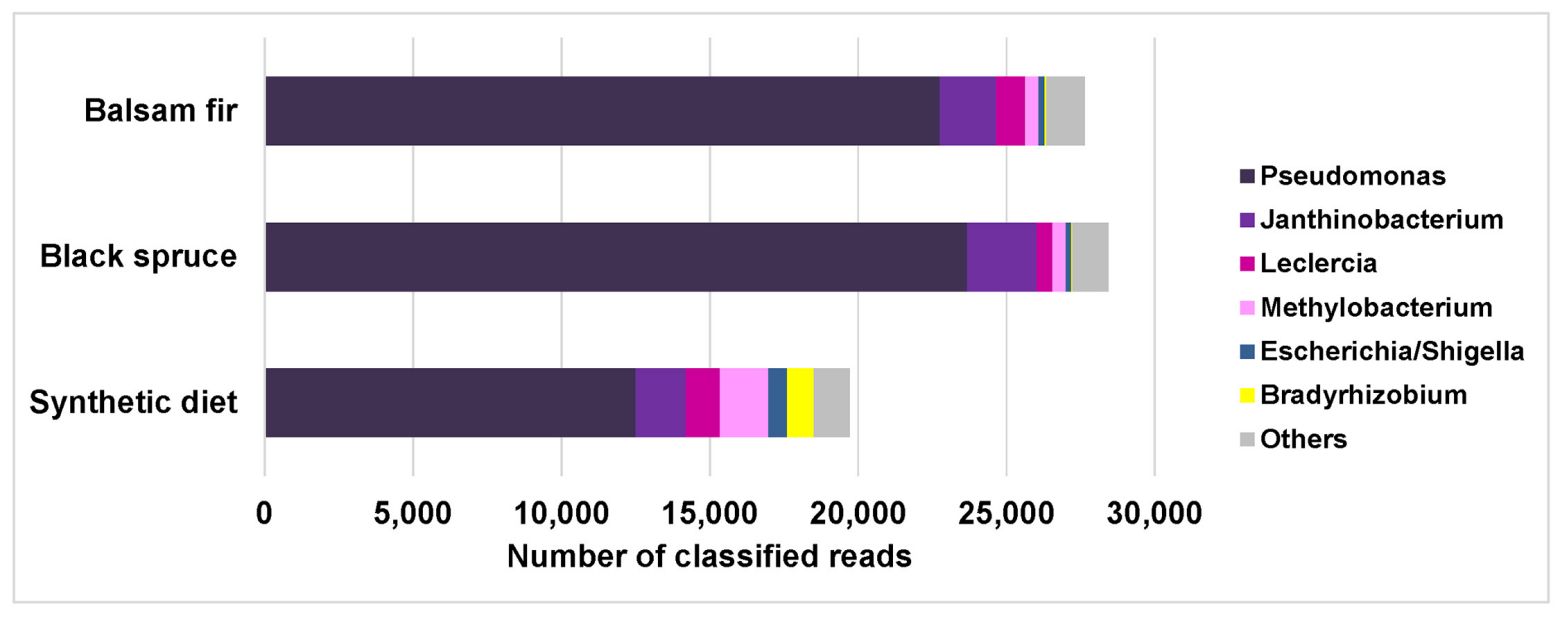
Taxonomic distribution of reads assigned to the Proteobacteria (taxonomic breakdown within purple bars of Fig 2) for each experimental group. Only genera comprising more than ~1% of the phylum are shown.

Overall, the budworm midgut microbiota was composed primarily of Proteobacteria (Fig 2). The dominant genus was *Pseudomonas*, which constituted the majority of the Proteobacteria (Fig 3), whereas *Bradyrhizobium* was essentially specific to larvae reared on synthetic diet (915 reads *vs* 30–62 reads for each of the other two diet groups). An examination of the top 10 OTUs within the three diet groups (Table 1) clearly showed that the genus *Pseudomonas* was indeed the most abundant taxon in the budworm midgut microbiota, as the majority of dominant OTUs belonged to this genus. These *Pseudomonas* OTUs could not be assigned to species using the NCBI 16S database, since each typically had several hits with an *E*-value of zero and an identity of 100%. This being said, species such as *P*. *fluorescens* and *P*. *paea* were often among these hits. Table 1 also shows that the communities were highly skewed with respect to OTU distribution, with the 10 top OTUs representing a very large proportion of the total reads obtained (~60–80%), indicating that the remaining OTUs represented low-abundance, less frequent taxonomic groups. Surprisingly, the two taxa–*Staphylococcus* and *Enterococcus*–that were identified in an earlier study [11] as the most abundant in the budworm midgut, using a bacterial culture approach, were among the lower abundance OTUs in this study, represented by 249 and 4 reads, respectively, among the 88,560 reads of the dataset. At the phylum level, the microbiotas of larvae fed either spruce or fir were very similar, except for a higher proportion of Actinobacteria (majority *Propionibacterium*) in larvae collected and reared on balsam fir (Fig 2; p < 0.01, Mann-Whitney). In larvae reared on artificial diet, the proportion of Bacteroidetes was much higher than in larvae collected and reared on the other two food types (p < 0.01, Kruskal-Wallis). More than 97% of these Bacteroidetes were assigned to the family Chitinophagacaea, but we could not assign them to specific genera using the GreenGenes database. However, some of the most abundant OTUs found in larvae fed artificial diet belonged to this family and were identified as *Hydrotalea flava* or *Sediminibacterium gingensoli* using the NCBI 16S database (Table 1).

**Table 1:** List of top 10 OTUs within each experimental group, along with the names of the species or genera they most likely represent (from BLAST analysis).

Group	OTU ID	Most likely species or genus	Reads	Abundance (%)
Balsam fir	Otu0348	*Pseudomonas sp*.	5584	18.9
	Otu0352	*Pseudomonas sp*.	4711	16.0
	Otu0354	*Pseudomonas sp*.	3767	12.8
	Otu0357	*Pseudomonas sp*.	3294	11.2
	Otu0361	*Pseudomonas sp*.	1697	5.7
	Otu0362	*Pseudomonas sp*.	1496	5.1
	Otu0365	*Pseudomonas sp*.	1226	4.2
	Otu0370	*Pseudomonas sp*.	684	2.3
	Otu1277	*Rugamonas rubra*	534	1.8
	Otu1278	*Rugamonas rubra*	456	1.5
***Total =***				**79.4**
Black spruce	Otu0346	*Pseudomonas sp*.	7315	24.8
	Otu0347	*Pseudomonas sp*.	7034	23.8
	Otu0359	*Pseudomonas sp*.	2244	7.6
	Otu0360	*Pseudomonas sp*.	1754	5.9
	Otu0364	*Pseudomonas sp*.	1295	4.4
	Otu0366	*Pseudomonas sp*.	1174	4.0
	Otu0368	*Pseudomonas sp*.	998	3.4
	Otu0371	*Pseudomonas sp*.	611	2.1
	Otu1279	*Rugamonas rubra*	444	1.5
	Otu0372	*Pseudomonas sp*.	441	1.4
***Total =***				**78.9**
Synthetic diet	Otu0351	*Pseudomonas sp*.	4566	15.5
	Otu0356	*Pseudomonas sp*.	3344	11.3
	Otu0358	*Pseudomonas sp*.	2191	7.4
	Otu0345	*Pseudomonas sp*.	1882	6.4
	Otu0517	*Hydrotalea flava*	1719	5.8
	Otu0519	*Sediminibacterium ginsengisoli*	834	2.8
	Otu1276	*Rugamonas rubra*	814	2.8
	Otu0521	*Sediminibacterium ginsengisoli*	751	2.5
	Otu0522	*Sediminibacterium ginsengisoli*	716	2.4
	Otu2190	*Methylobacterium aerolatum*	627	2.1
***Total =***				**59.1**

### Compositional variation among microbiotas (β-diversity)

**Fig 4:**
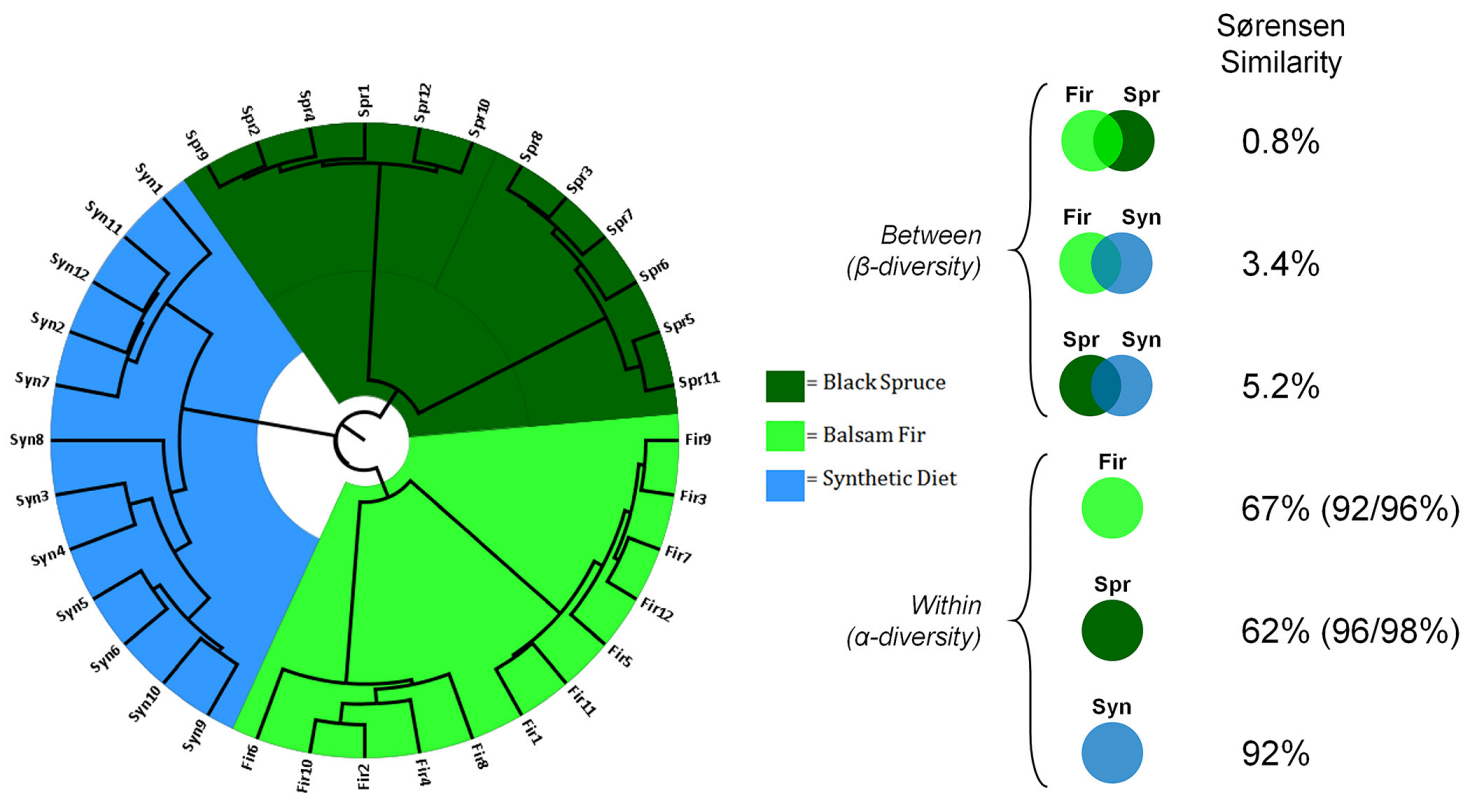
Individual and group diversity patterns. Dendrogram depicting the Bray-Curtis distances between each larva (left) and Sørensen similarities between or within the three groups (right). Note that individuals within the fir and spruce experimental groups form two subclades, each displaying high within subclade similarity (92–98%, in parentheses) compared to each group taken as a whole (62 and 67%).

The Bray-Curtis analysis, which is based on the presence/absence and relative abundance of OTUs in each sample, showed a strong clustering of individual larval microbiotas according to rearing conditions (Fig 4). Interestingly, the microbiotas of larvae reared on the two host trees clearly formed two compositional sub-clusters. To further compare the composition of the microbiotas among the three food sources, we calculated abundance-based Sørensen similarity values among and within the groups (Fig 4). The results of this analysis support the conclusions drawn from the Bray-Curtis similarity analysis, i.e., strong separation as a function of food source, with very little overlap (only ~1–5%) among diet groups. However, microbiotas of individual larvae reared on artificial diet all displayed very high similarity among one another (92%). For the two groups of larvae reared on host trees, the Sørensen similarity values calculated for each sub-cluster were higher than those calculated for the whole group, supporting the conclusion drawn from the Bray-Curtis analysis and pointing to the greater variation in the composition of the midgut microflora among larvae collected and reared on natural foliage.

## Discussion

The present study shows that the vast majority of bacteria found in the spruce budworm midgut belong to the phylum Proteobacteria (Fig 2), with the genus *Pseudomonas* constituting the dominant taxon, irrespective of rearing conditions (Fig 3). Indeed, more than 60% of the reads generated from larvae reared on artificial diet belonged to this genus, while this proportion increased > 80% in field-collected insects. A previous study using culture-dependent methods to identify bacteria from spruce budworm midguts also identified *Pseudomonas* as one of the taxa present in laboratory-reared insects, although in much lower proportions than those reported here [11]. This difference could result from taxon-specific differential growth of midgut bacteria on the tryptic soy agar medium used by these authors. Differential growth may also explain why the same study reported *Enterococcus* and *Staphylococcus* as the two dominant bacterial genera in budworm midguts, while these two Firmicute genera represented a low proportion of the culture-independent experiment reported here. It should also be pointed out that notable differences in the presence/absence of midgut bacterial taxa have been reported for different laboratory cultures of the same insect (e.g., *Enterobacter* in *Lymantria dispar*; [8,11]), suggesting the possibility of important differences in host genotypes controlling gut bacterial communities among different sources of laboratory-reared insects [17]. A study combining culture-dependent and culture-independent approaches for the characterization of the gypsy moth (*L*. *dispar*) midgut microbial community also identified *Pseudomonas* as representing a significant proportion of the microbiota [8]. *Pseudomonas fluorescens*, which is very closely related to the dominant bacterial OTUs in our samples, has been shown to degrade pectin, one of the components of plant cell walls [9]. Whether this bacterium plays a role in foliage digestion remains to be determined, but it could explain its possible dominance in the budworm midgut bacterial community.

The main difference between the communities of larvae reared in the laboratory and sampled in the field was the presence of a higher proportion of bacteria belonging to the phylum Bacteroidetes in insects reared on artificial diet (Fig 2). Prominent among these bacteria were the Chitinophagacea (more than 97% of Bacteroidetes reads), which could not be assigned to specific genera using the GreenGenes97 reference database; however, they were identified as *Hydrotalea flava* or *Sediminibacterium gingensoli* using the NCBI 16S database (see Table 1). These bacteria have also been reported in the gut of *Nyssomyia neivai* sandflies, which feed on the blood of other insects [18]. Their role, if any, in the biology of *C*. *fumiferana* is unknown, but their absence in field-collected insects suggests they are not essential and that they may be repressed by antibacterial compounds present in conifer leaves [12] or their growth may be favored in insects fed on artificial diet.

We found twice as many OTUs in larvae reared on artificial diet as compared to those collected in the field (Fig 1). Whether the presence of antimicrobial secondary metabolites in fir and spruce foliage is responsible for the lower taxonomic richness assessed in larvae collected on host plant materials is unclear at this point, as several other factors could account for this difference. Although the antibiotic present in the synthetic diet apparently did not inhibit midgut bacterial growth, it may nonetheless have affected the taxonomic composition of the intestinal microbiota. For example, rearing of budworms on an Aureomycin-laden diet over several generations may have led to the elimination of some bacterial taxa, leaving their niches open to the establishment of others. Similarly, the greater diversity of midgut bacteria in artificial-diet-reared insects could be an adaptive response to the diversity of components in this wheat-germ-based diet, thus favoring the growth of gut Bacteroidetes which have been associated with degradation of high-molecular-weight proteins and carbohydrates [19]. Finally, various other factors, including genetic differences between field-collected and laboratory-reared insects, could play a role in shaping the observed differences. An experiment involving the rearing of budworms from a single genetic stock on different diets over several generations would likely shed light on this issue. Interestingly, earlier studies focusing on lepidopteran midgut microbiota have reported a poorer microbial diversity in insects reared on artificial diet as compared to insects reared on natural host plants [20,21]. This discrepancy may simply be related to differences in experimental design, although the possibility remains that there are fir- and spruce-borne compounds that interfere with budworm midgut bacterial growth [12], whereas the opposite may be true for species where the insect’s gut microbiota depends largely on the microbial community present on the surface of the host plant [20].

In comparing the effects of diet on features of the budworm midgut microbiota, we observed a greater bacterial taxonomic similarity among larvae reared on artificial diet (92% Sørensen similarity), as compared to larvae collected on either host tree (62–67% Sørensen similarity; Fig 4). Although this difference could be due, at least in part, to the normalizing/homogenizing effects of Aureomycin (in the synthetic diet) and multi-generation rearing in the laboratory, it seems largely attributable to the presence of two clearly distinct Bray-Curtis clusters in each group of field-collected larvae (Fig 4). Since all larvae obtained from the field were processed in the same fashion, it appears that the deep within-host-tree taxonomic subdivision results from factors inherent to the larvae themselves. Given a sex ratio of ~50:50 in the spruce budworm [22] and an observed segregation of larvae in the same proportions within each host group (Fig 4), we speculate that the sex of the larvae (not recorded in this study) may be the factor responsible for the observed clustering.

Relatively minor differences were observed between the midgut microbiotas of larvae collected on balsam fir as opposed to those collected on black spruce, at least at the phylum (Fig 2) and genus (for Proteobacteria; Fig 3) levels. The main difference was a slightly greater proportion of Actinobacteria in the gut of larvae collected on balsam fir. Additional differences are likely present at the species level, as suggested by the presence of different phylotypes within each of these two diet groups (Table 1). However, the significance of such differences is unclear at this point and its assessment will require more extensive sampling of larvae on host trees from different geographical regions and habitats.

Interestingly, the present analysis suggested the absence, in the spruce budworm, of a distinct, core midgut microbiota, i.e., a set of bacterial taxa present in all processed larvae. Although some genera were present in all three diet groups (Table 1), none of the phylotypes were detected in all sampled larvae. This observation is in contrast with earlier studies where the authors reported the presence of a few bacterial species in all insects examined (e.g., [8,20]). It has been pointed out, however, that a lack of core gut microbiota among healthy individuals of a given species may not be critical so long as the microbial phylotypes present in the gut collectively provide a core set of bacterial genes required by the host [23], a situation that may apply to the spruce budworm gut microbiota.

In summary, the present work provides baseline data on the spruce budworm midgut microbiota, as determined using a culture-independent NGS approach. It also provides evidence that rearing conditions, including diet, can have a significant effect on budworm midgut microbiota species diversity. As such, the findings reported here should serve as a starting point for more in-depth studies examining the possible functions of the budworm intestinal microbiota; such functions could include processing of secondary plant metabolites, nutrition/digestion, immunity and communication, among others [4]. To this end, metagenomics and transcriptomics approaches will likely be required.

## Supporting Information

S1 Table Sequences of primers used to amplify the 16S V6-V8 region for pyrosequencing (from [13]). The dots are used to separate the different parts of the primers (Roche adaptor • multiplex identifier (barcode) • specific primer). Multiplex identifiers are only present in the forward primers, hence there is only one unique, shorter reverse primer.
(DOCX)
