# Supplementary material for: Composition of the Spruce Budworm (Choristoneura fumiferana) Midgut Microbiota as Affected by Rearing Conditions
Source: PLoS One. 2015 Dec 4;10(12):e0144077. doi: 10.1371/journal.pone.0144077 (PMC4670206; doi:10.1371/journal.pone.0144077)
Supplement: S1 Table — The dots are used to separate the different parts of the primers (Roche adaptor • multiplex identifier (barcode) • specific primer). Multiplex identifiers are only present in the forward primers, hence there is only one unique, shorter reverse primer. (DOCX) [file pone.0144077.s001.docx]

**Supporting information**

Table S1. Sequences of primers used to amplify the 16S V6-V8 region for pyrosequencing (from [13]). The dots are used to separate the different parts of the primers (Roche adaptor • multiplex identifier (barcode) • specific primer). Multiplex identifiers are only present in the forward primers, hence there is only one unique, shorter reverse primer.

| **Primer Name** | **Sequence 5’-3’** |
| --- | --- |
| A1-B969F | CCATCTCATCCCTGCGTGTCTCCGACTCAG•ACGAGTGCGT•ACGCGHNRAACCTTACC |
| A2-B969F | CCATCTCATCCCTGCGTGTCTCCGACTCAG•ACGCTCGACA•ACGCGHNRAACCTTACC |
| A3-B969F | CCATCTCATCCCTGCGTGTCTCCGACTCAG•AGACGCACTC•ACGCGHNRAACCTTACC |
| A4-B969F | CCATCTCATCCCTGCGTGTCTCCGACTCAG•AGCACTGTAG•ACGCGHNRAACCTTACC |
| A5-B969F | CCATCTCATCCCTGCGTGTCTCCGACTCAG•ATCAGACACG•ACGCGHNRAACCTTACC |
| A6-B969F | CCATCTCATCCCTGCGTGTCTCCGACTCAG•ATATCGCGAG•ACGCGHNRAACCTTACC |
| A7-B969F | CCATCTCATCCCTGCGTGTCTCCGACTCAG•CGTGTCTCTA•ACGCGHNRAACCTTACC |
| A8-B969F | CCATCTCATCCCTGCGTGTCTCCGACTCAG•CTCGCGTGTC•ACGCGHNRAACCTTACC |
| A9-B969F | CCATCTCATCCCTGCGTGTCTCCGACTCAG•TAGTATCAGC•ACGCGHNRAACCTTACC |
| A10-B969F | CCATCTCATCCCTGCGTGTCTCCGACTCAG•TCTCTATGCG•ACGCGHNRAACCTTACC |
| A11-B969F | CCATCTCATCCCTGCGTGTCTCCGACTCAG•TGATACGTCT•ACGCGHNRAACCTTACC |
| A12-B969F | CCATCTCATCCCTGCGTGTCTCCGACTCAG•TACTGAGCTA•ACGCGHNRAACCTTACC |
| B-BA1406R | CCTATCCCCTGTGTGCCTTGGCAGTCTCAG•ACGGGCRGTGWGTRCAA |
